# Strategy for Screening of Antioxidant Compounds from Two Ulmaceae Species Based on Liquid Chromatography-Mass Spectrometry

**DOI:** 10.3390/molecules23071830

**Published:** 2018-07-23

**Authors:** Joong Yeun Won, Su Young Son, Sunmin Lee, Digar Singh, Sarah Lee, Jong Seok Lee, Choong Hwan Lee

**Affiliations:** 1 Department of Bioscience and Biotechnology, Konkuk University, 120 Neungdong-ro, Gwangjin-gu, Seoul 05029, Korea; tomkazasky@naver.com (J.Y.W.); syson119@naver.com (S.Y.S.); duly123@naver.com (S.L.); singhdigar@gmail.com (D.S.); 2 National Institute of Biological Resources, Environmental Research Complex, Incheon 22689, Korea; lsr57@korea.kr (S.L.); jslee001@korea.kr (J.S.L.)

**Keywords:** *Aphananthe aspera*, *Zelkova serrata*, metabolite profiling, LC-MS, antioxidant activity, preparative HPLC combination

## Abstract

Liquid chromatography-mass spectrometry (LC-MS)-based untargeted metabolomics implies that annotated metabolites can serve as potential markers of the associated bioactivities of plant extracts. Firstly, we selected *Aphananthe aspera* and *Zelkova serrata* (Family: Ulmaceae) from 16 Korean plant species based on their distinct principal component analysis (PCA) patterns in LC-MS datasets and antioxidant activity assays. Further, we chose 40% solid-phase extraction (SPE) extracts of the two species displaying the highest antioxidant activities coupled with distinct PCA patterns. Examining the metabolite compositions of the 40% SPE extracts, we observed relatively higher abundances of quercetin, kaempferol, and isorhamnetin *O*-glucosides for *A. aspera*, whereas quercetin, isorhamnetin *O*-glucuronides, and procyanidin dimer were relatively higher in *Z. serrata*. These metabolites were clearly distinguished in pathway map and displayed strong positive correlations with antioxidant activity. Further, we performed preparative high-performance liquid chromatography (prep-HPLC) analysis coupled with the 2,2′-azino-bis (3-ethylbenzothiazoline-6-sulfonic acid) assay to validate their functional correlations. As a result, quercetin *O*-sophoroside was determined as the main antioxidant in *A. aspera*, while isorhamnetin *O*-glucuronide and procyanidin dimer were the primary antioxidants in *Z. serrata*. The current study suggests that the LC-MS-based untargeted metabolomics strategy can be used to illuminate subtle metabolic disparities as well as compounds associated with bioactivities.

## 1. Introduction

Many indigenous plant species are widely distributed on the Korean peninsula because of its distinct seasonal changes and heterogeneous terrains with unique features [1]. The indigenous plant species are often characterized to explore their diverse functional secondary metabolites, which engender distinct bioactivities and pharmacological effects including antioxidant, anti-inflammatory, and potential anticancer properties [2]. In particular, plants display various antioxidant mechanisms by engaging numerous secondary metabolites in order to protect themselves from oxidative damage caused by singlet oxygen, superoxide, and hydroxyl ions. Hence, the plant extracts rich in certain classes of secondary metabolites have also been traditionally revered for their pharmacotherapeutic effects and used as drug ingredients for treating various human ailments [3]. In recent years, plant metabolomics has emerged as an important tool for examining the gamut of natural bioactive products, with extensive applications for characterizing functional metabolites, drug research and development, as well as plant chemical diversity [4]. The metabolomics analyses commonly utilizes a range of analytical platforms such as gas chromatography-mass spectrometry (GC-MS), capillary electrophoresis-MS (CE-MS), liquid chromatography-MS (LC-MS) and nuclear magnetic resonance (NMR) spectroscopy [5]. In particular, LC-MS based metabolomics is considered as a widely used strategy for the relative comparison of both targeted and untargeted metabolite profiles as well as the screening of bioactive compounds owing its high sensitivity and selectivity coupled with rapid analysis ability [6]. Accordingly, it implies that the LC-MS based metabolite profiling may offer many advantages towards a high-throughput quantitative and qualitative analysis of plant secondary metabolites, thereby comprehending the plant metabolomics, associated phenotypes and bioactivities [7].

Approximately 230 species of the Ulmaceae family are distributed throughout North-East Asia, including Korea, China, Japan, and most other countries in the Northern Hemisphere [8]. The species of *Aphananthe aspera, Celtis koraiensis*, *Celtis sinensis*, *Hemiptelea davidii*, *Ulmus davidiana*, and *Zelkova serrata* are particularly abundant in Korea [9,10]. The stem-bark of *A. aspera* has been traditionally used for its medicinal values, including anti-inflammatory, anti-cancer, and anti-proliferative effects, as well as ache treatment [11,12]. The naive leaves of *Z. serrata* have culinary applications in certain foods (rice cakes) and beverages (tea), while its stem-bark and leaves have pharmacological applications because of their high abundance of antifungal, antioxidant, and anticancer compounds [13,14]. Notwithstanding the established phytochemical values of the Ulmaceae plant family, the detailed metabolite profiles and antioxidant compounds represented in its varying genera have largely remained uncharted. Accordingly, the present study was performed to investigate the LC-MS-based discriminant metabolite profiles for two Ulmaceae plant species prevalent in Korea, *A. aspera* and *Z. serrata*, simultaneously correlating their respective antioxidant activities in respective plant extracts. Herein, we propose a metabolomics approach that can be employed directly or used to supplement current methods toward the comprehensive analysis of secondary metabolites, applicable in studies including plant phytochemistry and natural product discovery, among many others.

## 2. Results and Discussion

In order to assess the metabolic variability among 16 indigenous plant species, we performed ultrahigh-performance liquid chromatography linear-trap quadrupole ion-trap tandem mass spectrometry (UHPLC-LTQ-IT-MS/MS) of the crude metabolite extracts, followed by multivariate statistical analyses. Notably, the principal component analysis (PCA) indicated that 14 species belonging to the plant families Amaranthaceae, Lamiaceae, Liliaceae, Violaceae, and Vitaceae were clustered according to their common phylogenetic traits, except the members of Ulmaceae family. The metabolic profiling datasets for the Ulmaceae members (*A. aspera* and *Z. serrata*) were clearly distinguished across PC1 (29.05%) (Figure S1A). The PCA model accuracy was evaluated with satisfactory statistical parameters, including “R^2^X,” which explains variance of X-data, and “Q^2^,” which illustrates the predicted variance. The PCA score plot showed the cumulative R^2^X (R^2^X _(cum)_) and Q^2^ (Q^2^_(cum)_) values of 41.1% and 18.5%, respectively. Further, we estimated the antioxidant activities for the 16 indigenous plant species using the 2,2′-azino-bis (3-ethylbenzothiazoline-6-sulfonic acid) (ABTS) radical scavenging assay and compared the bioactivities of the plant families, using the average values for species representing each family. Significantly higher antioxidant activities were observed for five representative species in the following order: *V. ficifolia* > *P. tricuspidata* > *A. aspera* > *Z. serrata* > *V. coignetiae* (Figure S1B). However, significantly higher average antioxidant values were observed for Ulmaceae and Vitaceae with marginal differences, followed by Lamiaceae, Amaranthaceae, Liliaceae, and Violaceae (Figure S1C). Hence, we conjecture that despite the environmental variables among the samples (harvest locations and times), the phylogenetic relatedness based on the metabolic compositions of most of the samples, except for the two Ulmaceae species, remained unaltered as inferred through the PCA (Table S1). This inspired further study, intended to delineate the metabolic disparities and screen for potent antioxidant compounds between the two Ulmaceae species.

**Figure 1:**
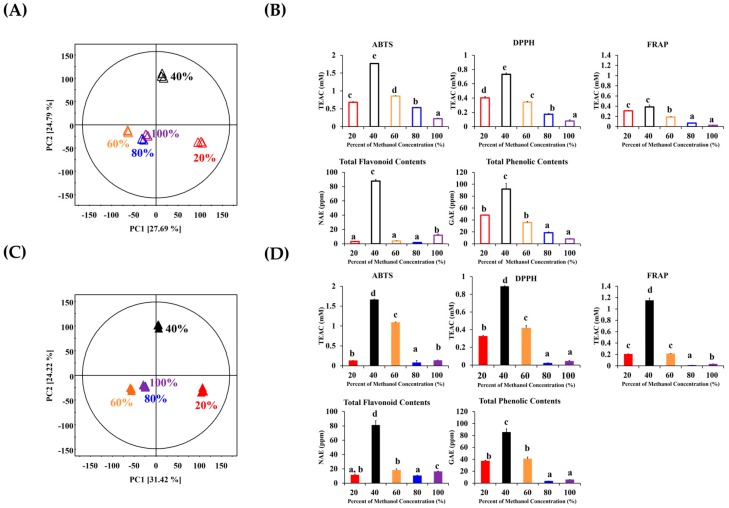
Principal component analysis (PCA) score plot (**A**) and (**C**) from UHPLC-LTQ-IT-MS/MS; antioxidant activity assays using ABTS, DPPH radical scavenging, FRAP, TFC, and TPC assays (**B**) and (**D**) derived from stepwise SPE extracts of *A. aspera* (unfilled column) and *Z. serrata* (filled column): *red column* 20% SPE extract, *black column* 40% SPE extract, *orange column* 60% SPE extract, *blue column* 80% SPE extract, and *violet column* 100% SPE extract.

We used the stepwise solid-phase extraction (SPE) strategy to screen the potent antioxidant compounds of the two Ulmaceae species. The purified stepwise SPE extracts were analyzed by UHPLC-LTQ-IT-MS/MS followed by multivariate statistical analysis and antioxidant activity assays (ABTS and 2,2-diphenyl-1-picrylhydrazyl (DPPH) radical scavenging activity assays), ferric reducing ability of plasma (FRAP) reducing-power assay, and assays for the total phenolic contents (TPC) as well as total flavonoid contents (TFC). Herein, 40% methanol SPE extracts of *A. aspera* and *Z. serrata* displayed distinctly separate patterns along PC2 (24.79% and 24.22%) in the PCA (Figure 1A,C) and simultaneously indicated relatively higher antioxidant activities than the other SPE extracts (Figure 1B,D).

**Figure 2:**
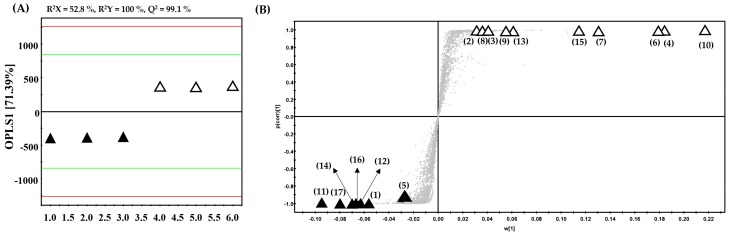
Multivariate statistical analysis of LC-MS datasets for 40% methanol SPE extracts of *A. aspera* (unfilled triangles) and *Z. serrata* (filled triangles): (**A**) OPLS-DA score plot, with VIP >1.0 and *p*-value <0.05 marked; (**B**) loading S-plot based on OPLS-DA. Numbers labeling each sample′s discriminant metabolites on the plot correspond to entries in Table 1.

**Figure 3:**
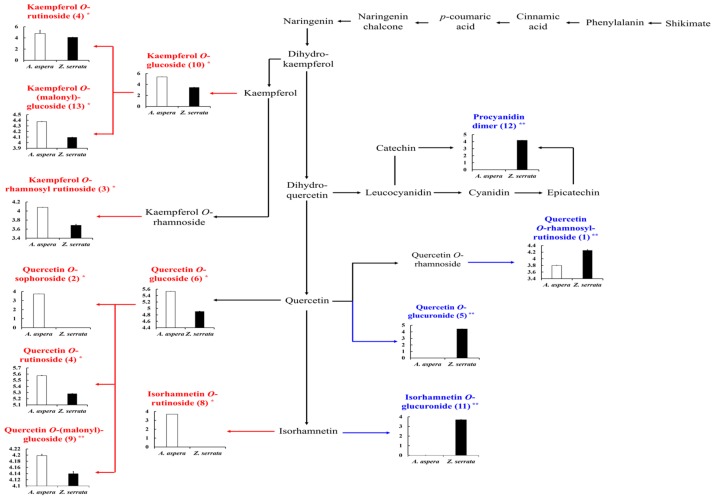
Discriminant metabolic pathway. The unfilled and filled columns represent the relative abundance of discriminant metabolites between *A. aspera* and *Z. serrata*. The scheme of the pathway is derived from the KEGG database (KEGG, http://www.genome.jp/kegg). The Y-axis of the graph indicates peak areas at logarithmic scale. The data are presented as the mean ± standard deviation. Differences were considered significant at *p*-value < 0.05. The metabolite numbers in parentheses are those presented in Table 1. * The discriminant metabolites in *A. aspera* are indicated with red font ** and those in *Z. serrata* are indicated with blue font.

The partially purified (40% SPE extracts) samples were further analyzed using UHPLC-LTQ-IT-MS/MS, followed by multivariate statistical analysis and ultra-performance liquid chromatograph quadrupole-time-of-flight MS (UPLC-Q-TOF-MS) methods to examine the distinct metabolic entities having significantly higher antioxidant activities. The LC-MS-based orthogonal partial-least-squares discriminant analysis (OPLS-DA) plots (Figure 2A) displayed an overall 71.39% variance along OPLS1 with satisfactory R^2^X (52.8%), R^2^Y (100%), and Q^2^ (99.1%) values. Further, an S-plots of OPLS-DA based on the loading was constructed (Figure 2B) to discover and visualize significant variables contributing to the differentiation of the selected SPE extracts among the two Ulmaceae species. In the S-plot, longer distances from the midpoints of the selected variables signify higher metabolic disparity between the two species. Accordingly, we determined a total of 17 metabolites as largely discriminant contributing to the maximum metabolomic disparity between the two Ulmaceae species at VIP > 1.0 and *p*-value < 0.05. The discriminant metabolites were putatively identified based on their retention times, mass to charge ratios (*m*/*z*), MS^n^ fragment patterns, and UV λ_max_ (nm) data obtained through UHPLC-LTQ-IT-MS/MS analysis, as well as by annotations using an in-house library and literature [15,16,17,18,19]. In addition, mass errors (mDa) and elemental composition data obtained from the UPLC-Q-TOF-MS analysis data are used to support the putative metabolite identities (Table 1). Further, we presented a scheme of the metabolic pathways describing the biosynthetic routes of discriminant metabolites (Figure 3); unfilled and filled columns indicate their relative abundance in *A. aspera* and *Z. serrata*, respectively. Notably, the 40% SPE extracts displayed a significant disparity with respect to *O*-glucosylated isorhamnetin and quercetin (**2**, **4**, **6**, **8**, and **9**) and for the *O*-glycosylation of kaempferol (**3**, **7**, **10**, and **13**), with relatively higher abundance in *A. aspera*. Meanwhile, quercetin *O*-rhamnosyl rutinoside (**1**), isorhamnetin and quercetin *O*-glucuronides (**5** and **11**), and procyanidin dimer (**12**) levels were relatively higher in *Z. serrata*. We conjecture that the varying degrees of glycosylation of flavonols contribute maximally to the observed disparity between the Ulmaceae plant species. Previously, various quercetin and kaempferol derivatives were reported to be present in *Z. serrata*; hence, these compounds could serve as important biomarkers toward annotating discriminant metabolites among the Ulmaceae species [20,21]. In addition, it was reported that aglycones varied with respect to the degree of glycosylation and localization within plant tissues in different species, and thus could influence their physiochemical traits [22]. Considering these studies, we propose that *O*-glycosylated kaempferol, glucosylated isorhamnetin, and quercetin are highly abundant in *A. aspera* extracts, whereas glucuronidated quercetin, isorhamnetin, and procyanidin dimer levels are significantly higher in *Z. serrata* extracts. Based on this, we can propose that sugar derivatives of the flavonols seem to significantly affect the observed metabolic disparities between the two examined Ulmaceae species.

**Figure 4:**
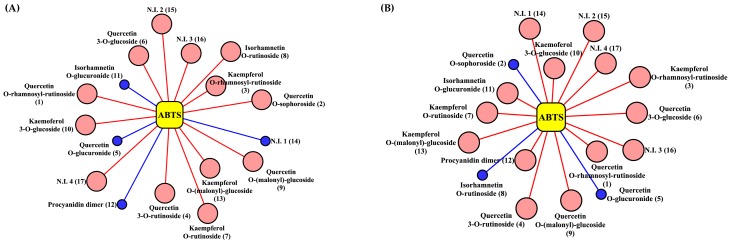
Visualization of the correlation networks between the identified secondary metabolites and ABTS radical scavenging activity assay of *A. aspera* (**A**) and *Z. serrata* (**B**) according to Pearson′s correlation coefficient. Correlation coefficients higher than 0.80 or lower than −0.80 with *p*-value < 0.05 are extracted and shown in these association networks. Each node indicates an identified metabolite and corresponding ABTS assay. Yellow node = antioxidant activity assay, Pink node = positively correlated metabolite, Blue node = negatively correlated metabolite. The sizes of the nodes represent the degrees of association. The numbers on the metabolites are those indicated in Table 1.

In order to examine the correlations between the relative abundance of metabolites and the observed antioxidant activities for extracts from plants of different species, we constructed correlation networks. The correlation analysis was performed based on statistical operations using the relative peak areas for selected compounds and the observed trolox equivalent antioxidant capacity (TEAC) intensities obtained via the ABTS radical scavenging assay. In the correlation network (Figure 4), lines connect the metabolites and ABTS assay, signifying correlation coefficients either greater than *r* > 0.80 or lower than *r* < −0.80 at *p*-value < 0.05. Notably, 10 metabolites overall—quercetin *O*-rhamnosyl rutinoside (**1**), quercetin *O*-sophoroside (**2**), kaempferol *O*-rhamnosyl rutinoside (**3**), quercetin *O*-rutinoside (**4**), quercetin *O*-glucoside (**6**), kaempferol *O*-rutinoside (**7**), isorhamnetin *O*-rutinosiode (**8**), quercetin *O*-(malonyl)-glucoside (**9**), kaempferol *O*-glucoside (**10**), and kaempferol *O*-(malonyl)-glucoside (**13**)—were positively correlated with antioxidant activity, whereas the remaining three metabolites (quercetin *O*-glucuronide (**5**), isorhamnetin *O*-glucuroside (**11**), and procyanidin dimer (**12**)) had negative correlations in *A. aspera*. Meanwhile, three metabolites—quercetin *O*-sophoroside (**2**), quercetin *O*-glucuronide (**5**), and isorhamnetin *O*-rutinoside (**8**)—exhibited negative correlations, while the remaining 10 metabolites showed high positive correlations with antioxidant activity in *Z. serrata*.

**Figure 5:**
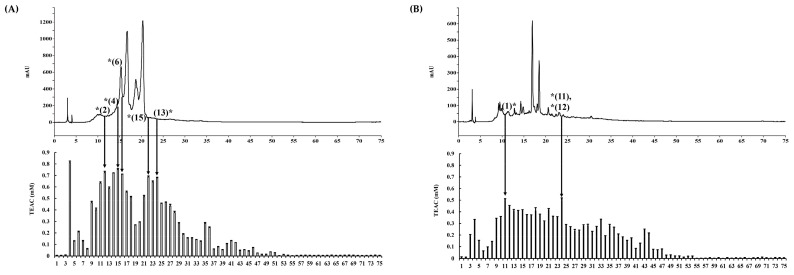
Preparative HPLC profiles, ABTS radical scavenging assay of *A. aspera* (unfilled columns) and *Z. serrata* (filled columns) extracts: (**A**) *A. aspera* preparative HPLC fractions at 300 nm; (**B**) *Z. serrata* preparative HPLC fractions at 300 nm, Data are shown as mean ± standard deviation; The number of the fractions indicate to Table 1 suggested metabolite number. * Selected fraction, which is contributed to relative potent antioxidant activity.

**Figure 6:**
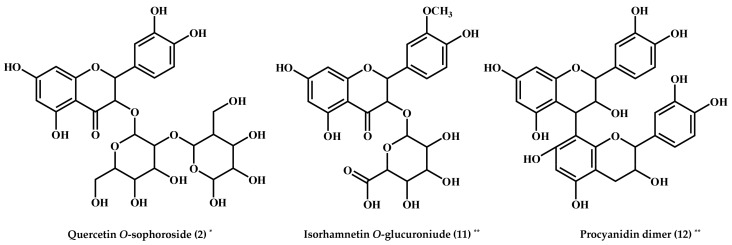
The structures of compounds selected in prep-HPLC fractions from *A. aspera* and *Z. serrata* extracts with potent antioxidant activities. * Main antioxidant compound in *A. aspera*; ** Main antioxidant compounds in *Z. serrata*. The number of the fractions, indicated in Table 1, suggest the metabolite number.

In the networks, we found that the correlation tendencies between the two species are slightly different. Hence, we further examined the major antioxidant metabolites in the partially purified 40% SPE extracts from *A. aspera* and *Z. serrata* using preparative HPLC (Prep-HPLC), followed by ABTS assay for the collected fractions. Prep-HPLC analysis is commonly employed as a metabolite isolation strategy because of its outstanding separation and purification efficiency. A total of 75 fractions of each sample were acquired, followed by ABTS assays of each of the two sample extracts. Further, the selected fractions with considerably higher antioxidant activities were subjected to UPLC-Q-TOF-MS analysis to identify their metabolite components. In this step, we primarily identified quercetin *O*-sophoroside (**2**) from 12 min, quercetin *O*-rutinoside (**4**) from 15 min, quercetin *O*-glucoside (**6**) from 16 min, non-identified compound 2 (N.I. 2) (**15**) in 22 min, and kaempferol *O*-(malonyl)-glucoside (**13**) in 24 min prep-HPLC fractions of the *A. aspera* SPE extracts (Figure 5A). Quercetin *O*-rhamnosyl rutinoside (**1**) in 11 min and isorhamnetin *O*-glucuronide (**11**) and procyanidin dimer (**12**) in 24 min fractions were identified as the strong antioxidant candidate metabolites in *Z. serrata* contributing to the metabolic disparity (Figure 5B). Previously, it was reported that the antioxidant activity of the extracts was influenced by the proportional contents of the metabolites, as displayed statistically through the correlation analysis [23]. Specifically, quercetin *O*-sophoroside (**2**) displayed a positive correlation with ABTS antioxidant activity only in *A. aspera* extracts, and thus was chosen as the strong antioxidant compound in the prep-HPLC fractions. On the other hand, isorhamnetin *O*-glucuronide (**11**) and procyanidin dimer (**12**) were selected as the strong antioxidants in *Z. serrata* extracts with positive correlations to the ABTS activity. Structurally, the quercetin *O*-sophoroside (**2**) is a quercetin derivative of *O*-sophoroside known for its antioxidant activities. As shown in Figure 6, the specific OH− groups at the 3′ and 4′ carbons of the B-ring and the 3 and 4 carbons of the C-ring engender the strong antioxidant effects of quercetin *O*-sophoroside [24,25]. The isorhamnetin *O*-glucuronide (**11**) structure consists of *O*-methylated quercetin linked with *O*-glucuronoside, where the B-ring OH− groups also transfer hydrogen atoms and electrons to hydroxyl, peroxyl, and peroxynitrite radicals [25]. The procyanidin dimer (**12**) belongs to the family of proanthocyanidins and is composed of (−)-epicatechin or (+)-catechin units, linked through C4→C8 of the two flavan-3-ol groups, yielding a characteristics inter-flavonoid linkage [26]. Similar to the first three compounds, the antioxidant activity of the proanthocyanidins is also affected by the number of OH− groups scavenging the free radicals [27]. In addition, the inter-flavonoid C4→C8 bond reportedly enhances the antioxidant properties of procyanidin dimers [28]. Hence, it can be stated that the selected three metabolites are the principally different variables contributing to the potent antioxidant activities in each of the two Ulmaceae species; they may contribute to the subtle metabolic disparities between these closely related plant species.

The application of the proposed and demonstrated systematic LC-MS-based metabolomics approach can greatly facilitate identification of relatively strong antioxidant activity-contributing compounds between the selected Ulmaceae species. Recapitulating the results, seventeen discriminant metabolite variables between the two species were identified by LC-MS analysis and subsequent screening by prep-HPLC coupled with antioxidant activity assays. In conclusion, we could understand that activity-guided systematic characterization of untargeted metabolites using LC-MS-based metabolomics has potential applications in the identification of candidate metabolites that induce metabolic disparities among closely related species. Further, it is expected that the proposed methodology can be employed toward the rapid examination of bioactive compounds in indigenous plant species with possible ethnopharmacological applications.

## 3. Materials and Methods

### 3.1. Chemicals and Reagents

HPLC-grade water, ethanol, methanol, and acetonitrile were purchased from Fisher Scientific (Pittsburgh, PA, USA). Analytical-grade potassium persulfate, 2,2-azino-bis (3-ethylbenzothiazoline -6-sulfonic acid) diammonium salt (ABTS), 1,1-diphenyl-2-picrylhydrazyl (DPPH), hydrogen chloride (HCl), 2,4,6-tris(2-pyridyl)-*S*-triazine (TPTZ), iron (III) chloride, sodium acetate, acetic acid, Folin-Ciocalteu′s phenol reagent, formic acid, and standard compounds (HPLC-grade) 6-hydroxy-2,5,7,8-tetramethylchroman-2-carboxylic acid (Trolox), gallic acid, naringin, quercetin 3-*O*-glucoside (purity ≥ 98.0%), quercetin 3-*O*-rutinoside (purity ≥ 94.0%), and kaempferol 3-*O*-glucoside (purity ≥ 97.0%) were purchased from Sigma-Aldrich (St. Louis, MO, USA). Sodium carbonate and diethylene glycol were obtained from Junsei Chemical Co., Ltd. (Tokyo, Japan).

### 3.2. Sample Information and Preparation

Sixteen indigenous Korean plant species leaves from different families including Amaranthaceae (three), Ulmaceae (two), Lamiaceae (four), Liliaceae (two), Violaceae (two), and Vitaceae (three) were examined in this work (Table S1). The samples were procured from the National Institute of Biological Resources (NIBR, Incheon, Korea). All samples representing differen species belonging to the six named plant families were first dried under shade, followed by the extraction of each sample (100 mg) with 70% ethanol (1 L). The leaf extracts were then concentrated using a rotary evaporator (N-1000SWD, Eyela, Tokyo, Japan) at 45 °C for 24 h and filtered. Before conducting experiments, the samples were extracted immediately and stored under deep-freeze conditions (−70 °C). The extracts for LC-MS analyses were dissolved in 1 mL of 70% ethanol through sonication for 5 min at 4 °C (Hettich Zentrifugen Universal 320, Tuttlingen, Germany) and then filtered through a 0.2-μm polytetrafluoroethylene (PTFE) syringe filter. The filtered samples were completely dried using a speed-vacuum concentrator (Biotron, Seoul, Korea), and the dried extracts were reconstituted in 70% ethanol (10 mg/mL) prior to LC-MS analyses. The quality control (QC) samples were made by using the pooled mixture (60 μL) from each sample (biological replicate) to ascertain the operational or instrumental errors potentially generated during the LC-MS analyses.

### 3.3. LC-MS Analysis

The UHPLC-LTQ-IT-MS/MS analysis was performed according to the method previously described by Lee et al. (2015) [2]. The analysis was conducted using an LTQ XL ion-trap mass spectrometer (Thermo Fisher Scientific, Bremen, Germany) coupled with a Dionex UHPLC system (Dionex Corporation, Sunnyvale, CA, USA) consisting of an UltiMate 3000 RS (Rapid Separation) Pump, an UltiMate 3000 RS Autosampler, and an UltiMate RS 3000 Column Compartment with a Thermostable Column area and an UltiMate RS 3000 Diode Array Detector (Dionex Corp., Sunnyvale, CA, USA). Each sample (10 μL) was injected into a Thermo Scientific Syncronis C18 UHPLC column (100 mm length × 2.1 mm inner diameter, × 1.7-μm particle size) at the flow rate of 0.3 mL/min; the column temperature was retained at 35 °C. Ion trapping was analyzed by electrospray ionization in negative ion mode, positive ion mode, and full-scan ion modes within the mass range 150–1000 *m*/*z*. The operating parameters were set to source voltage at ±5 kV, capillary voltage at ± 39 kV, and capillary temperature at 275 °C. Tandem MS (MS/MS) analysis was performed by scan-type turbo data-dependent scanning (DDS) under the same conditions used for MS scanning. The photodiode array detector was set to scan the wavelength range 200–600 nm and managed by a 3D field.

For ultra-performance liquid chromatograph quadrupole-time-of-flight mass spectrometry (UPLC-Q-TOF-MS) analysis, we followed a method partially adapted from Lee et al. (2015) [2]. The analysis was performed on an UPLC ACQUITY system (Waters, Milford, MA, USA), equipped with a binary solvent delivery apparatus, an auto-sampler, and an ultraviolet (UV) detector coupled to a Waters Q-TOF Premier MS (Micromass MS Technologies, Manchester, UK) system. The mobile phase consisted of solvent A (0.1% formic acid in water) and solvent B (0.1% formic acid in acetonitrile). The analytical sample (injection volume: 5 μL) was separated in an ACQUITY BEH C18 column (100 mm × 2.1 mm × 1.7 μm particle size; Waters Corp., Milford, MA, USA) at the flow rate of 0.3 mL/min and the column temperature of 37 °C. The MS data were collected in the range 100–1000 *m*/*z* using a Waters Q-TOF Premier system (Micromass MS Technologies, Manchester, UK) under negative and positive ion modes. The desolvation gas (nitrogen) was set to 650 L/h at a temperature of 300 °C. The cone gas (nitrogen) was set to 50 L/h, and the source temperature was 80 °C. The capillary and cone voltages were set to 2.5 kV and 30 V, respectively. Data were collected in the centroid mode with a scan accumulation time of 0.2 s. All analyses were performed using an independent reference spray via the LockSpray interference to ensure lock mass [*m*/*z* 554.2615 (negative ion mode) and *m*/*z* 556.2771 (positive ion mode)] at a flow rate of 10 μL/min. Accurate masses and elemental compositions were calculated using the MassLynx software (Waters Corp., Milford, MA, USA) incorporated in the instrument.

### 3.4. Solid Phase Extraction and Prep-HPLC Separation

The solid-phase extraction (SPE) procedure was performed using the modified method described previously by Hamedeyazdan et al. (2017) [29] and Betthauser et al. (2017) [30]. In this experiment, SPE was performed for 70% ethanol extracts (2 g) from *A. aspera* and *Z. serrata* leaves, and the quantified extracts were re-constituted in 70% ethanol (700 μL, 1,400,000 ppm). Prior to the elution, each C18 reversed-phase cartridge (Sep-Pak 20 cc, 5g, Waters Corp., Milford, MA, USA) was pre-activated using 50 mL distilled water and 10% aqueous methanol solution to remove any impurities in the cartridge. The methanol solution of each sample was fractionated on the cartridge using a stepwise methanol-water gradient mixture (20:80, 40:60, 60:40, 80:20, and 100:0) of 50 mL, generating five fractions. Following the partial separation on the Sep-Pak cartridge, the collected fractions (*n* = 5) for each sample were dried using a rotary evaporator at 30 °C and speed vacuum concentrator at 30 °C.

Prep-HPLC separation was performed using a modified method (Kuang et al., 2013) [31]. The prep-HPLC equipment used was a Hitachi LaChrom Elite Organizer with an L-2130 pump (Hitachi, Tokyo, Japan) and L-2455 diode array detector (Hitachi, Tokyo, Japan) with a reversed-phase C18 column (4.6 mm × 250 mm, 5 μm particle size; YMC Corp., Kyoto, Japan). The mobile phase consisted of solvent A (5% aqueous acetonitrile) and solvent B (100% acetonitrile). The gradient program was set as follows: 5% solvent B was maintained initially for 2 min followed by a gradual increase to 100% over 73 min and then maintained at 5% solvent B for 2 min. The total run time was 75 min. To acquire the fractions of the two samples, the methanol solution of each SPE extract (40 mg) was injected into the prep-HPLC column phase at the optimal flow rate (1 mL/min). A total of 75 fractions of each SPE extract were purified in this experiment. The UV detection wavelength was 400 nm for monitoring each extract. Ultimately, all 75 fractions of each SPE extract were collected manually and dried using a rotary evaporator at 30 °C.

### 3.5. Determination of Antioxidant Activity Using ABTS, DPPH, and FRAP Assays

ABTS antioxidant activity was performed using the method suggested by Lee et al. [5]. The stock solution included ABTS stock solution (7 mM) and potassium persulfate stock solution (2.45 mM). The working solution was prepared by mixing the two stock solutions in equal quantities and allowing them to react for 12 h at room temperature in the dark. To process with the analysis, we found the optimal absorbance of 0.70 ± 0.02 at 750 nm using a microplate reader by diluting the ABTS solution (Thermo Electron, Spectronic Genesys 6, Madison, WI, USA). The 16 indigenous species resolved in 70% ethanol solution, SPE extracts, and 75 prep-HPLC fractions for each *A. aspera* and *Z. serrata* were dried and reconstituted in 100% methanol prior to the antioxidant activity assays. The radical scavenging activity was measured by adding 20 μL of each sample to 180 μL of diluted ABTS solution in the wells of a 96-well microtiter plate, and the reaction was incubated at 37 °C for 6 min in the dark. Then, the absorbance was assessed at 750 nm using a microplate reader. The radical scavenging activity was measured in triplicates and the standard curve was observed as linear between 0.0078 mM and 2 mM TEAC. The results were expressed in millimolar TEAC per milligram of the dry weight of the SPE extract.

The DPPH assay was carried out following the method adapted from Lee et al. (2015) [2]. The five stepwise SPE extracts from *A. aspera* and *Z. serrata* were dissolved with 100% methanol solution. For the DPPH radical scavenging assay, 180 μL of the DPPH stock solution (0.2 mM in ethanol) was mixed with 20 μL of the two species′ SPE stepwise extracts, respectively, in 96-well plates and reacted for 20 min at room temperature in the dark. The DPPH free radical absorbance was measured at 515 nm using a microplate reader. The radical scavenging activity was measured in triplicate and the standard curve was linear between 0.0078 mM and 1 mM TEAC.

The FRAP assay was measured as described previously by Son et al. (2016) [22] with some modifications. The five stepwise SPE extracts for *A. aspera* and *Z. serrata* were reconstituted in 100% methanol solution. The FRAP reagents were mixed with an acetate buffer (pH 3.6 in distilled water), 10 mM TPTZ (in 40 mM HCl solution), and 20 mM FeCl_3_·6H_2_O (in distilled water) at a ratio of 10:1:1. Accordingly, 10 μL of each sample extract was mixed with 300 μL of the FRAP solvent in the 96-well microtiter plates, and the reaction was incubated for 6 min at 37 °C in the dark. The absorbance was recorded using the microplate reader at 570 nm. The radical scavenging activity was measured for three biological as well as analytical replicates, and the standard curve was observed as linear between 0.0078 mM and 2 mM of TEAC.

### 3.6. Determination of TPC and TFC

The TPCs were examined using the methods adapted from Jung et al. (2013) [32]. The five stepwise SPE extracts of the crude leaf solvent extracts from *A. aspera* and *Z. serrata* were reconstituted in 100% methanol. In a 96-well microtiter plate, 20 μL of each sample and 100 μL of 0.2 N Folin-Ciocalteu’s phenol reagent were mixed, and the reaction was incubated in the dark for 5 min at room temperature. Immediately after, 80 μL of a 7.5% sodium carbonate solution was added to the reaction mixture, and the mixture was incubated for 60 min at room temperature. Finally, the reaction absorbance was measured at 750 nm. The TPCs were expressed as gallic acid equivalent concentrations (ppm); results were reported as the mean of three replicates. The standard solution concentration curve ranged between 31.25 ppm and 500 ppm.

To measure the TFCs, we employed the method described previously by Lee et al. [5]. The five stepwise SPE leaf extracts from *A. aspera* and *Z. serrata* were reconstituted in 100% methanol. The reaction mixture was made by adding 180 μL of 90% diethylene glycol (in distilled water), 20 μL of 1 N NaOH, and 20 μL of sample into the wells of the 96-well microtiter plate, and the reaction was incubated for 60 min at room temperature in the dark. The reaction absorbance was recorded at 405 nm. The TFCs were expressed as naringin equivalent concentrations (ppm) and data were reported as the mean of three biological as well as analytical replicates. The standard solution concentration curve ranged between 15.625 ppm and 200 ppm.

### 3.7. Data Processing and Statistical Analysis

The UHPLC-LTQ-IT-MS/MS data were acquired with Xcalibur software (version 2.00, Thermo Fisher Scientific) and the raw data files were converted to NetCDF (*.cdf) format using Xcalibur software. The UPLC-Q-TOF-MS data were obtained with MassLynx software (version 4.1, Waters Corp.). Then, the raw data files were converted to NetCDF (*.cdf) format using the MassLynx DataBridge (version 4.1, Waters Corp.). After conversion, the UHPLC-LTQ-IT-MS/MS and UPLC-Q-TOF-MS NetCDF files were subjected to preprocessing corrections for retention time, baseline extraction, and peak extraction using the Metalign software package (http://www.metalign.nl). The resulting data were exported to Microsoft Excel (Microsoft, Redmond, WA, USA). Multivariate statistical analysis was performed using SIMCA-P + 12.0 software (Umetrics, Umea, Sweden). The principal component analysis (PCA), orthogonal partial least-square discriminant analysis (OPLS-DA), and loading S-plot interpretations were performed to examine the metabolic disparities between the studied plant species. The metabolic variables were selected based on the variable importance in the projection (VIP) values and the significant differences were determined by analysis of variance (ANOVA). After the multivariate statistical analyses, significantly discriminant metabolites were putatively identified using in-house libraries, the chemical dictionary version 7.2 (Chapman and Hall/CRC), references, and standard compounds through comparing both the mass spectra and retention times. Pairwise correlations between selected secondary metabolites of 40% SPE extracts of the two species and antioxidant activity (ABTS) were calculated by Pearson′s correlation coefficient test using PASW Statistics 18; the correlation network analyses were visualized by Cytoscape software (http://www.cytoscape.org/).

## Figures and Tables

**Table 1:** Identification of tentative compounds to be used as variables to classify 40% SPE extracts of *A. aspera* and *Z. serrata* on the basis of LC-MS results.

No.	Tentative Identifications ^a^	UHPLC-LTQ-ESI-IT-MS/MS					UPLC-Q-TOF-MS				I.D. ^e^
		t_R_^b^(min)	Measured Mass (*m*/*z*)		[M − H]^−^MS ^n^ Fragments (*m*/*z*) ^c^	UV (nm)	Measured Mass (*m*/*z*) [M − H]^−^	Elemental Composition [M − H]^−^	mDa	i-FIT (norm) ^d^	
			[M − H]^−^	[M + H]^+^							
**Flavonoids**											
1	Quercetin *O*-rhamnosyl rutinoside **	6.60	755	757	755 > 737, 609, 591, 489, 343, 301, 271	267sh, 366sh ^f^	755.2042	C33H39O20	0.7	0.102	Ref. [15]
2	Quercetin *O*-sophoroside *	6.62	625	627	625 > 505, 463, 445, 301, 271, 255	266sh, 366sh	625.1405	C27H29O17	0.4	2.215	Ref. [16]
3	Kaempferol *O*-rhamnosyl-rutinoside *	7.09	739	741	739 > 693, 593, 575, 393, 285, 255	256, 355	739.1663	C33H39O19	0.6	0.726	Ref. [15]
4	Quercetin *O*-rutinoside *	7.45	609	611	609 > 301 > 273, 257, 179, 151	256, 354	609.1456	C27H29O16	−2.0	1.482	LIB
5	Quercetin *O*−glucuronide **	7.68	477	479	477 > 301 > 273, 257, 179 > 151	281, 316sh	477.0669	C21H17O13	0.3	0.260	Ref. [15,19]
6	Quercetin *O*-glucoside *	7.69	463	465	463 > 301 > 273, 257, 179 > 151	265, 346, 366sh	463.0877	C21H19O12	−0.1	0.258	LIB
7	Kaempferol *O*-rutinoside *	7.85	593	595	593 > 285 > 267, 257, 229, 213, 179, 163	266, 346	593.1506	C27H29O15	1.1	0.171	Ref. [15,16,17]
8	Isorhamnetin *O*-rutinoside *	7.94	623	625	623 > 315 > 300, 287 > 271, 255	281, 366sh	623.1612	C28H31O16	−0.7	0.523	Ref. [15,17,19]
9	Quercetin *O*-(malonyl)-glucoside *	7.99	549	551	549 > 505 > 463, 301 > 273, 257, 179, 151	281, 381sh	549.0880	C24H21O15	0.4	0.261	Ref. [17]
10	Kaempferol *O*-glucoside *	8.12	447	449	447 > 327, 285 > 267, 257, 241 > 239, 229, 163	281, 334sh	447.0927	C21H19O11	0.1	1.485	LIB
11	Isorhamnetin *O*-glucuronide **	8.36	491	493	491 > 315 > 300 > 271, 255, 151	280, 325sh	491.0826	C22H19O13	−0.1	0.341	Ref. [18]
12	Procyanidin dimer **	8.43	575	577	575 > 449, 437, 394, 287	281, 319sh	575.1190	C30H23O12	−0.2	2.055	Ref. [19]
13	Kaempferol *O*-(malonyl)-glucoside *	8.46	533	535	533 > 489 > 285 > 267, 257, 229, 197, 163	279sh, 327sh	533.0931	C24H21O14	0.1	1.343	Ref. [17]
**Non-Identified**											
14	N.I. 1 **	7.36	567	569	567 > 521, 405, 359, 341, 329	256, 354	567.2078	C27H35O13	1.9	2.412	-
15	N.I. 2 *	8.27	451	453	451 > 341, 299 > 323, 297, 231, 217, 177	270, 351sh	451.1026	C24H19O9	−0.3	1.235	-
16	N.I. 3 **	8.57	625	627	625 > 607, 540, 463, 445, 415, 397, 227	281	625.2708	C27H45O16	0.0	0.325	-
17	N.I. 4 **	8.77	551	553	551 > 389, 329, 227	281	551.2336	C24H39O14	−0.4	2.612	-

^a^ Tentatively identified metabolites based on both VIP > 1.0, *p*-value < 0.05 and OPLS1 by OPLS-DA dataset; ^b^ RT, Retention time; ^c^ MS^n^ fragment patterns detected in negative mode; ^d^ i-FIT (norm) is a measure of how well the observed isotope pattern matches the predicted isotope pattern for the formula on that line; ^e^ I.D., identification; LIB, In-house library; Ref., References. ^f^ sh, Shoulder; * Mainly detected compound in *A. aspera*; ** Mainly detected compound in *Z. serrata*.

## Supplementary Materials

The Supplementary Materials are available online.

## References

[1] Kim Y.S. (2006). Conservation of plant diversity in Korea. Landsc. Ecol. Eng..

[2] Lee S., Oh D.G., Lee S., Kim G.R., Lee J.S., Son Y.K. (2015). Chemotaxonomic metabolite profiling of 62 indigenous plant species and its correlation with bioactivities. Molecules.

[3] Tohma H., Gülçin İ., Bursal E., Gören A.C., Alwasel S.H., Köksal E. (2017). Antioxidant activity and phenolic compounds of ginger (Zingiber officinale Rosc.) determined by HPLC-MS/MS. J. Food Meas. Charact..

[4] Raterink R.J., Lindenburg P.W., Vreeken R.J., Ramautar R., Hankemeier T. (2014). Recent developments in sample-pretreatment techniques for mass spectrometry-based metabolomics. Trends Anal. Chem..

[5] Lee M.Y., Singh D., Kim S.H., Lee S.J., Lee C.H. (2016). Ultrahigh Pressure Processing Produces Alterations in the Metabolite Profiles of Panax ginseng. Molecules.

[6] Kumar S., Chandra P., Bajpai V., Singh A., Srivastava M., Mishra D.K., Kumar B. (2015). Rapid qualitative and quantitative analysis of bioactive compounds from *Phyllanthus amarus* using LC/MS/MS techniques. Ind. Crop. Prod..

[7] Lim D.K., Mo C., Lee J.H., Long N.P., Dong Z., Li J. (2018). The integration of multi-platform MS-based metabolomics and multivariate analysis for the geographical origin discrimination of *Oryza sativa* L.. J. Food Drug Anal..

[8] Zuo L.H., Shang A.Q., Zhang S., Yu X.Y., Ren Y.C., Yang M.S. (2017). The first complete chloroplast genome sequences of *Ulmus* species by de novo sequencing: Genome comparative and taxonomic position analysis. PLoS ONE.

[9] Lim J.C., Choi B.K., Kim S.Y., Eom B.C., Kim J.W. (2016). Korean traditional village forest (Ma-Eul-Soop) and potential natural vegetation: A case study on the Sachon-Ri Garo-Soop in Gyeongsangbuk-do, South Korea. J. Plant Biol..

[10] Yang M.Q., Li D.Z., Wen J., Yi T.S. (2017). Phylogeny and biogeography of the amphi-Pacific genus *Aphananthe*. PLoS ONE.

[11] Ye G., Fan M., Huang C. (2007). Ellagic acid glycosides from the stem bark of *Aphananthe aspera*. Chem. Nat. Comp..

[12] Sun J., Gao Q., Li X.B., Tang F., Li C.X. (2017). Antiproliferative constituents from *Aphananthe aspera* leaves. Phytochem. Lett..

[13] Lee H.Y., Kwon J.T., Koh M., Cho M.H., Park S.B. (2007). Enhanced efficacy of 7-hydroxy-3-methoxycadalene via glycosylation in in vivo xenograft study. Bioorg. Med. Chem. Lett..

[14] Kang H.J., Jang Y.J. (2012). Selective apoptotic effect of *Zelkova serrata* twig extract on mouth epidermoid carcinoma through p53 activation. J. Oral. Sci..

[15] Farag M.A., Sakna S.T., El-fiky N.M., Shabana M.M., Wessjohann L. (2015). Phytochemical, antioxidant and antidiabetic evaluation of eight Bauhinia, L. species from Egypt using UHPLC–PDA–qTOF-MS and chemometrics. Phytochemistry.

[16] Roldan M.V.G., Engel B., de Vos R.C., Vereijken P., Astola L., Groenenboom M. (2014). Metabolomics reveals organ-specific metabolic rearrangements during early tomato seedling development. Metabolomics.

[17] Lin L.Z., Harnly J.M. (2008). Phenolic compounds and chromatographic profiles of pear skins (*Pyrus* spp.). J. Agric. Food Chem..

[18] La Barbera G., Capriotti A.L., Cavaliere C., Piovesana S., Samperi R., Chiozzi R.Z., Laganà A. (2017). Comprehensive polyphenol profiling of a strawberry extract (Fragaria × ananassa) by ultra-high-performance liquid chromatography coupled with high-resolution mass spectrometry. Anal. Bioanal. Chem..

[19] D′Urso G., Pizza C., Piacente S., Montoro P. (2018). Combination of LC–MS based metabolomics and antioxidant activity for evaluation of bioactive compounds in *Fragaria vesca* leaves from Italy. J. Pharm. Biomed. Anal..

[20] Giannasi D.E. (1978). Generic relationships in the Ulmaceae based on flavonoid chemistry. Taxon.

[21] Santamour F.S. (1983). Flavonoid distribution in *Zelkova* [landscape tree in the United States]. J. Arbori..

[22] Stobiecki M., Skirycz A., Kerhoas L., Kachlicki P., Muth D., Einhorn J. (2006). Profiling of phenolic glycosidic conjugates in leaves of Arabidopsis thaliana using LC/MS. Metabolomics.

[23] Son S.Y., Kim N.K., Lee S., Singh D., Kim G.R., Lee J.S., Lee C.H. (2016). Metabolite fingerprinting, pathway analyses, and bioactivity correlations for plant species belonging to the Cornaceae, Fabaceae, and Rosaceae families. Plant Cell Rep..

[24] Zhang Y., Wang D., Yang L., Zhou D., Zhang J. (2014). Purification and characterization of flavonoids from the leaves of *Zanthoxylum bungeanum* and correlation between their structure and antioxidant activity. PLoS ONE.

[25] Heim K.E., Tagliaferro A.R., Bobilya D.J. (2002). Flavonoid antioxidants: Chemistry, metabolism and structure-activity relationships. J. Nutr. Biochem..

[26] Esatbeyoglu T., Wray V., Winterhalter P. (2015). Isolation of dimeric, trimeric, tetrameric and pentameric procyanidins from unroasted cocoa beans (*Theobroma cacao* L.) using countercurrent chromatography. Food Chem..

[27] Mendoza–Wilson A.M., Castro-Arredondo S.I., Balandrán-Quintana R.R. (2014). Computational study of the structure–free radical scavenging relationship of procyanidins. Food Chem..

[28] Da Silva Porto P.A.L., Laranjinha J.A.N., de Freitas V.A.P. (2003). Antioxidant protection of low density lipoprotein by procyanidins: Structure/activity relationships. Biochem. Pharm..

[29] Hamedeyazdan S., Niroumand F., Fathiazad F. (2017). Phytochemical analysis and antioxidative properties of Centaurea albonitens. Res. J. Pharma..

[30] Betthauser T.J., Ellison P.A., Murali D., Lao P.J., Barnhart T.E., Furumoto S., Christian B.T. (2017). Characterization of the radiosynthesis and purification of [18F] THK-5351, a PET ligand for neurofibrillary tau. Appl. Radiat. Isot..

[31] Kuang P., Song D., Yuan Q., Yi R., Lv X., Liang H. (2013). Separation and purification of sulforaphene from radish seeds using macroporous resin and preparative high-performance liquid chromatography. Food Chem..

[32] Jung E.S., Lee S., Lim S.H., Ha S.H., Liu K.H., Lee C.H. (2013). Metabolite profiling of the short-term responses of rice leaves (Oryza sativa cv. Ilmi) cultivated under different LED lights and its correlations with antioxidant activities. Plant Sci..

