# Supplementary material for: Strategy for Screening of Antioxidant Compounds from Two Ulmaceae Species Based on Liquid Chromatography-Mass Spectrometry
Source: Molecules. 2018 Jul 23;23(7):1830. doi: 10.3390/molecules23071830 (PMC6100396; doi:10.3390/molecules23071830)
Supplement: Supplementary file 1 [file molecules-23-01830-s001.pdf]

molecules

Supplementary Materials

Article

A Strategy for Screening of Antioxidant Compounds from Two Ulmaceae Species based on Liquid Chromatography Mass Spectrometry (LC-MS)

Joong Yeun Won [1], Su Young Son [1], Sunmin Lee [1], Digar singh [1] and Choong Hwan Lee [1,*]

[1] Department of Bioscience and Biotechnology, Konkuk University, 120 Neungdong-ro, Gwangjin-gu, Seoul 05029, Korea; tomkazasky@naver.com (J.W.); syson119@naver.com (S.S.); duly123@naver.com (S.L.); singhdigar@gmail.com (D.S.)

* Correspondence: Choong Hwan Lee (C.L.), chlee123@konkuk.ac.kr, Tel.: +82-2-444-4290 (C.L.)

Received: date; Accepted: date; Published: date

Table S1. Used sample information in this study.

No.	Family	Taxon	Collected location in Korea	Collected date
1	Amaranthaceae	*Celosia argentea*	Yongsan-ri, Buan-myeon, Gochang-gun, Jeollabuk-do	2015-09-18
2		*Achyranthes japonica*	Yeondong-ri, Imhoe-myeon, Jindo-gun, Jeollanam-do	2015-08-26
3		*Celosia cristata*	Gugok-ri, Bongyang-eup, Jecheon-si, Chungcheongbuk-do	2015-08-15
4	Ulmaceae	*Aphananthe aspera*	Guanum-do, Cheonbu-ri, Buk-myeon, Ulleung-gun, Gyeongsangbuk-do	2015-09-02
5		*Zelkova serrata*	Oji-ri, Ogok-myeon, Gokseong-gun, Jeollanam-do	2015-06-03
6	Lamiaceae	*Isodon japonicus*	Gyeseong-ri, Hanam-myeon, Hwacheon-gun, Gangwon-do	2015-09-08
7		*Phlomis umbrosa*	Donggeom-ri, Gilsang-myeon, Ganghwa-gun, Incheon	2015-08-12
8		*Agastache rugosa*	Myeong-wol-ri, Sanae-myeon, Hwacheon-gun, Gangwon-do	2015-08-11
9		*Lamium amplexicaule*	Daebuhdo, Daebuhdo-dong, Danwon-gu, Ansan-si, Gyeonggi-do	2015-04-06
10	Liliaceae	*Hemerocallis fulva*	Papo-ri, Sangseo-myeon, Hwacheon-gun, Gangwon-do	2015-08-12
11		*Smilacina japonica*	Bangtaesan, Bangdong-ri, Girin-myeon, Inje-gun, Gangwon-do	2015-05-27
12	Violaceae	*Viola verecunda*	Bangtaesan, Bangdong-ri, Girin-myeon, Inje-gun, Gangwon-do	2015-05-06
13		*Viola mandshurica*	Chundang-ri, Cheongil-myeon, Hoengseong-gun, Gangwon-do	2015-04-15
14	Vitaceae	*Vitis coignetiae*	Namseo-ri, Seo-myeon, Ulleung-gun, Gyeongsangbuk-do	2015-09-02
15		*Vitis ficifolia*	Seokjeong-ri, Geumsan-myeon, Goheung-gun, Jeollanam-do	2015-08-14
16		*Parthenocissus tricuspidata*	Nambuk-ri, Inje-eup, Inje-gun, Gangwon-do	2016-10-08

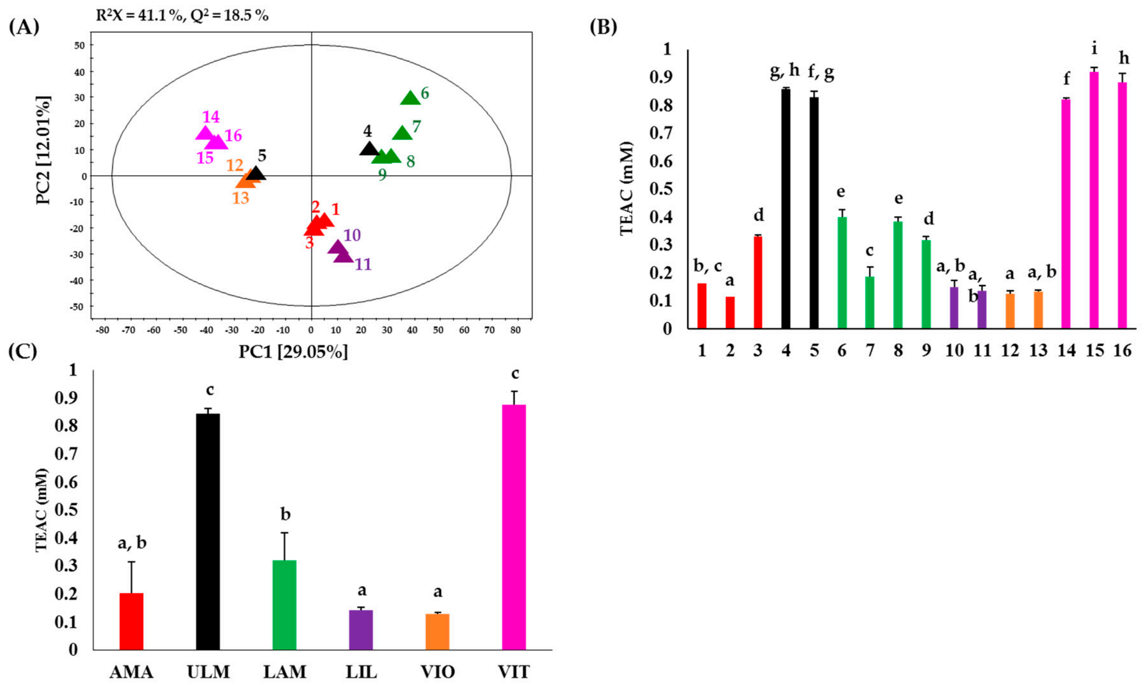

Figure S1. Mutivariate statistical analysis and antioxidant activity for 16 indigenous plant species. (A) PCA score plot based on LC-MS datasets; (B) ABTS radical scavenging activity assay; (C) Average ABTS radical scavenging activities observed for each family; Here, AMA: Amaranthaceae, ULM: Ulmaceae, LAM: Lamiaceae, LIL: Liliaceae, VIO: Violaceae, VIT: Vitaceae. The plant species indicated with numbers (1-16) are elaborated and enlisted in Table 1. Bar indicates the standard deviation. Different letters indicates statistical differences among the observed values based Duncan`s multiple range tests (p-value < 0.05), 'a', 'b', 'c', 'd', 'e', 'f', 'g', 'h' and 'i' describe statistically different.
